# Supplementary material for: An Optimization Framework for Silicon Photonic Evanescent-Field Biosensors Using Sub-Wavelength Gratings
Source: Biosensors (Basel). 2022 Oct 8;12(10):840. doi: 10.3390/bios12100840 (PMC9599562; doi:10.3390/bios12100840)
Supplement: Supplementary file 1 [file biosensors-12-00840-s001.zip › biosensors-1929082-supplementary.pdf]

Supplementary Materials

An Optimization Framework for Silicon Photonic Evanescent-Field Biosensors Using Sub-Wavelength Gratings

Lauren S. Puumala [1,2,*], Samantha M. Grist [1,2,3], Kithmin Wickremasinghe [4], Mohammed A. Al-Qadasi [4,†], Sheri Jahan Chowdhury [4,†], Yifei Liu [4], Matthew Mitchell [3,5], Lukas Chrostowski [3,4,5], Sudip Shekhar [3,4] and Karen C. Cheung [1,2,4,*]

[1] School of Biomedical Engineering, University of British Columbia, 251-2222 Health Sciences Mall, Vancouver, BC V6T 1Z3, Canada
[2] Centre for Blood Research, University of British Columbia, 2350 Health Sciences Mall, Vancouver, BC V6T 1Z3, Canada
[3] Dream Photonics Inc., Vancouver, BC V6T 0A7, Canada
[4] Department of Electrical and Computer Engineering, University of British Columbia, 5500-2332 Main Mall, Vancouver, BC V6T 1Z4, Canada
[5] Stewart Blusson Quantum Matter Institute, University of British Columbia, 2355 East Mall, Vancouver, BC V6T 1Z4, Canada
* Correspondence: lpuumala@student.ubc.ca (L.S.P.); kcheung@ece.ubc.ca (K.C.C.)
† These authors contributed equally to this work.

Section S1. Material Model Fitting for FDTD Simulations

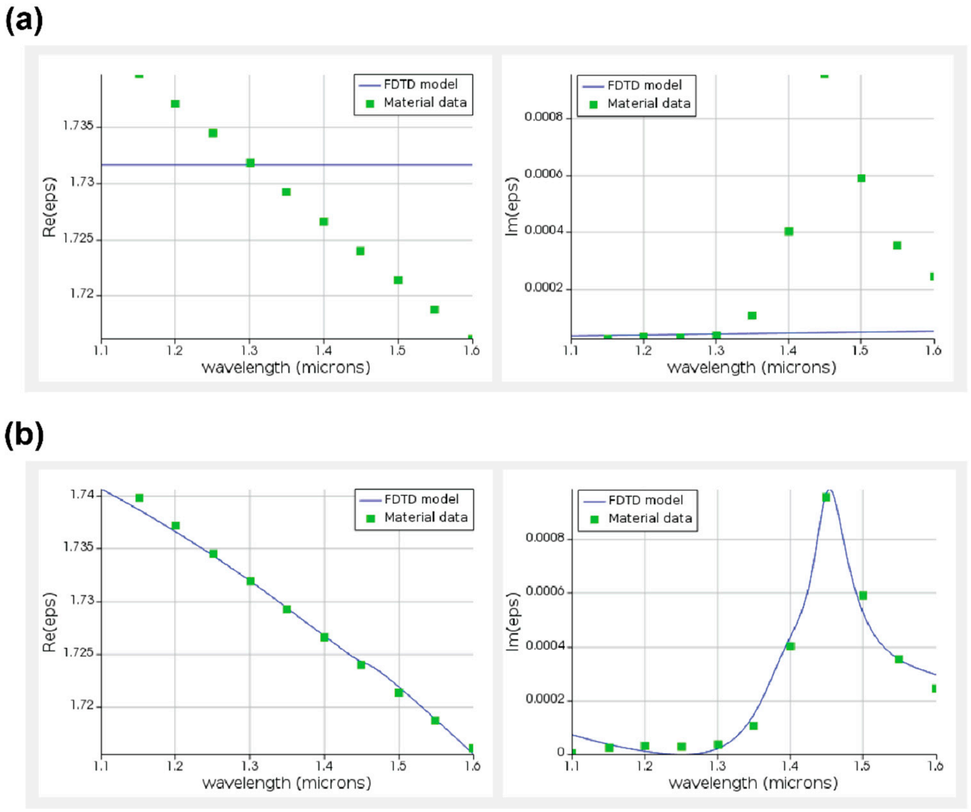

Figure S1. (**a**) Default multi-coefficient material model fit for the real (left) and imaginary (right) refractive indices of water (fit tolerance = 0.1, max coefficients = 6, and imaginary weight = 1). (**b**) Improved multi-coefficient material model fit for the real (**left**) and imaginary (**right**) refractive indices of water (fit tolerance = 1×10^{-6}, max coefficients = 10, and imaginary weight = 100).

Section S2. Microfluidic Setup

Prior to the bulk refractive index sensing experiments, two pieces of 0.02″ ID Tygon® microbore tubing (Saint-

Gobain Performance Plastics ND 100-80 AAD04103, Malvern, PA, USA) with blunt 22G needles (McMaster-Carr 75165A682, Elmhurst, IL, USA) attached on one end were inserted into the microfluidic outlets of the PDMS gasket. The microfluidic channels were pre-wet with ethanol, and injected into the gasket at the inlet ports using a syringe with a 22G blunt needle.

A two-channel modular Fluigent LineUp™ series fluid control system (Fluigent, Le Kremlin-Bicêtre, France), controlled by OxyGEN software (SSFT-OXY), was used to control and monitor the delivery of salt solutions to both channels of the microfluidic gasket mounted to the photonic chip. This system consists of a FLPG Plus pressure source and LineUp™ modules including the LINK (LU-LNK-002) software control module, two Flow EZ™ (LU-FEZ) pressure-based flow controllers, two M-SWITCH™ (ESSMSW003) 10-position bi-directional valves, a SWITCH-EZ (ELUSEZ) microfluidic valve controller, and two FLOW UNIT (model M) bidirectional flow rate sensors. The salt solutions were stored in ten 15-mL Falcon® tube (Corning Inc., Corning, NY, USA) reservoirs (five reservoirs for each channel) equipped with Fluigent P-CAP airtight metal caps that were connected to the air pressure output of each flow controller via 4 mm OD tubing and a 10-position manifold (Fluigent) to facilitate pressurization, and to the fluidic inlets of the bidirectional valves via 1/16″ OD, 0.010″ ID FEP capillary tubing (Fluigent). The outlets of the two bidirectional valves were connected to the inlets of the two flow sensors using the same FEP tubing. The flow sensor outlets were each connected to bubble traps (Diba Omnifit® #006BT-HF, Cole-Parmer Canada, Quebec, QC, Canada). The bubble trap outlets were connected to PEEK 1/32″ OD, 0.010″ ID capillary tubing (Idex 1531B, Cole-Parmer Canada, Quebec, QC, Canada), which was used to supply fluid to the photonic chip assembly.

Section S3. FDTD Simulations Modeling Incomplete Waveguide Wetting

The FDTD band structure simulation method described in Section 2.1.1 was used to estimate n_g and S_b for a fishbone SWG waveguide with incomplete wetting. The simulations were performed for design C1, using the as-fabricated waveguide dimensions that were measured from SEM images ($w = 478$ nm, $t = 220$ nm, $\Lambda = 250$ nm, $\delta = 0.475$, and $w_{fb} = 172$ nm). Air was added in the gaps between the silicon blocks of the SWG, replacing the background material in those regions (Figure S2). In these simulations, n_{eff}, n_g, and $\partial n_{eff} / \partial n_{bulk}$, were extracted, while sweeping the height of the air pocket (t_{air}) from 100 nm to 220 nm. S_b was calculated from these results.

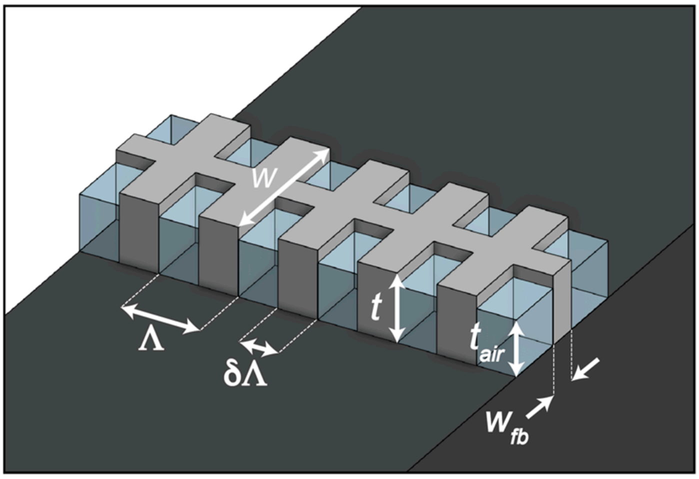

Figure S2. Illustration of the FDTD simulation setup used to investigate the effect of incomplete wetting on n_g and S_b for fishbone SWG design C1. The waveguide geometry used in this simulation was based on measurements made on SEM images of the fabricated waveguide ($w = 478$ nm, $t = 220$ nm, $\Lambda = 250$ nm, $\delta = 0.475$, and $w_{fb} = 172$ nm). The gaps between the SWG blocks were filled with air up to a height t_{air}. The same FDTD region, override mesh, simulation boundaries, light source, monitors, and background materials described in Section 2.1.1 were used in this simulation but are not illustrated here for simplicity.

As shown in Figure S3, the results of these simulations illustrate that air entrapment decreases n_g for the analyzed fishbone SWG structure. An increase in the height of the air pocket decreases n_g. In these simulations, the value of n_g intersects with the experimentally measured n_g for this structure when $t_{air} \approx 120$ nm. These simulation results suggest that air entrapment, combined with the decreased feature sizes of the fabricated waveguides, could be responsible for the discrepancy in n_g between the initial simulations and experimentally measured values.

As the height of the air pocket increases, S_b decreases due to a decrease in susceptibility. For the air pocket heights investigated here, the S_b values are somewhat lower than the experimentally measured values.

This simulation model does not account for the curvature of the air-water interfaces between the trapped air and bulk fluid. It also does not account for the slightly rounded corners of the SWG waveguides, which can be seen in the SEM images, or the presence of a native oxide layer on the silicon. Taken together, these factors could account for the remaining differences between the simulated and experimental sensitivity values.

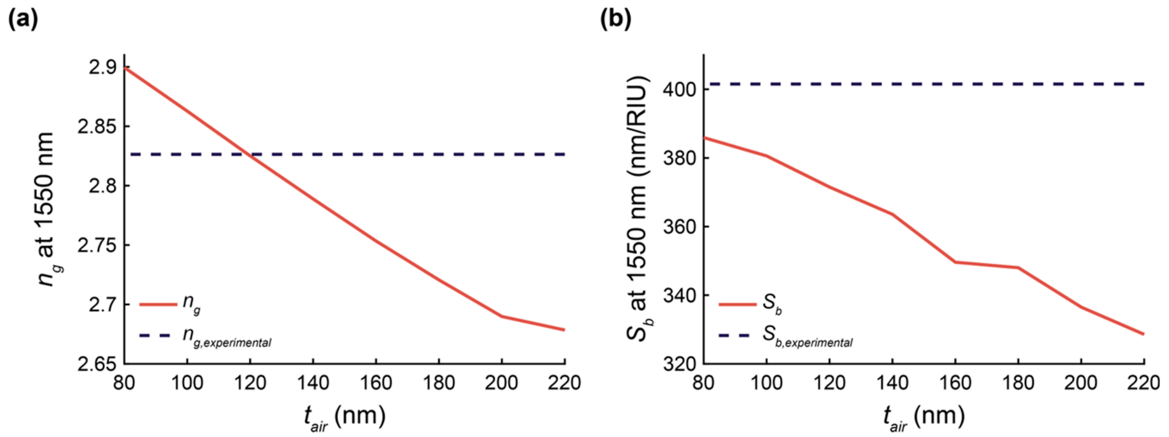

Figure S3. (**a**) Simulated group index, n_g, at 1550 nm versus air pocket height, t_{air}, for fishbone SWG waveguide C1 with as-fabricated geometry (w = 478 nm, t = 220 nm, Λ = 250 nm, δ = 0.475, and w_{fb} = 172 nm). The experimental n_g is also plotted as a dashed line. (**b**) Simulated bulk refractive index sensitivity, S_b, at 1550 nm versus air pocket height, t_{air}, for fishbone SWG waveguide C1 with as-fabricated geometry (w = 478 nm, t = 220 nm, Λ = 250 nm, δ = 0.475, and w_{fb} = 172 nm). The experimental S_b, averaged across both microfluidic channels, is also plotted as a dashed line. The simulations were performed using a 3D-finite difference time domain (FDTD) approach with water as the cladding material.

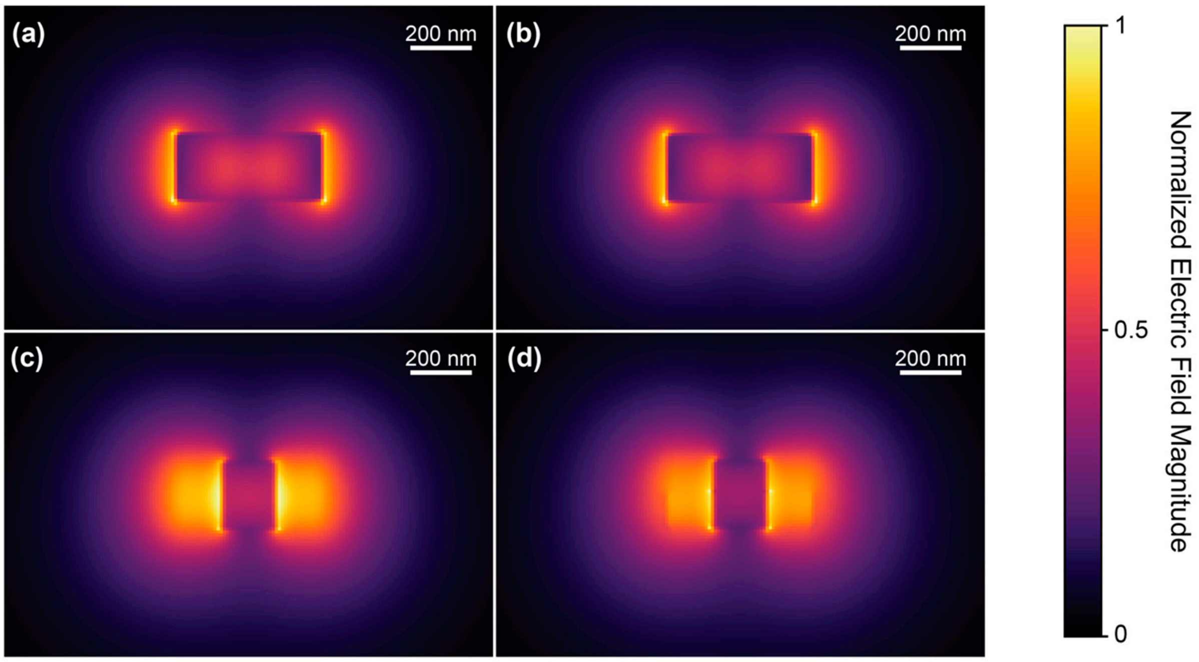

Figure S4. Cross-sectional electric field profiles for fishbone SWG waveguide C1 (as-designed geometry: w = 478 nm, t = 220 nm, Λ = 250 nm, δ = 0.475, and w_{fb} = 172 nm) obtained from 3D-FDTD simulations at 1550 nm without air entrapment (**a**,**c**) and with air entrapment (**b**,**d**).The top two subplots (**a**,**b**) show the cross-sectional field profile in the center of the SWG block, while the bottom two subplots (**c**,**d**) show the cross-sectional field profile in the center of the fishbone. An air pocket thickness of t_{air} = 120 nm was used for mode profiles (**b**) and (**d**). The data presented in all subplots has been normalized to the same maximum electric field intensity.

Section S4. Resonance Peaks Included in Extinction Ratio and Quality Factor Mean Calculations

Table S1. Number of resonances included in calculations to obtain the mean extinction ratios and quality factors and their standard deviations for each resonator design. For designs C3, C4, O3, and O4, two replicate resonators were fabricated for each coupling gap on each chip. For all other designs, one resonator was fabricated for each coupling gap on each chip. All resonances included in these calculations passed the $R^2 > 0.85$ threshold during the analysis described in Section 2.4. Variations in the number of included resonances between different devices and replicates can be at-

tributed to fabrication yield, damage prior to testing, noise, losses, split peaks, differences in *FSR*, and differences in the analyzed wavelength ranges.

MRR design	g_c (nm)	Number of analyzed resonances										Total
		Chip 1		Chip 2		Chip 3		Chip 4		Chip 5		
		Ring 1	Ring 2	Ring 1	Ring 2	Ring 1	Ring 2	Ring 1	Ring 2	Ring 1	Ring 2	
C1[ǂ]	450	7	-	5	-	5	-	5	-	4	-	26
	500	6	-	7	-	6	-	7	-	7	-	33
	550	0	-	6	-	7	-	7	-	3	-	23
	600	6	-	6	-	6	-	7	-	5	-	30
C2[ǂ]	450	6	-	6	-	6	-	6	-	6	-	30
	500	6	-	6	-	6	-	6	-	6	-	30
	550	5	-	6	-	6	-	6	-	6	-	29
	600	6	-	6	-	6	-	6	-	6	-	30
C3[ǂ]	400	7	7	7	7	7	7	7	7	7	6	69
	450	7	7	7	7	7	6	7	7	7	7	69
	500	7	7	7	7	7	6	3	7	7	7	65
	550	6	6	7	7	7	7	7	7	7	7	68
C4	450	6	6	5	6	6	7	1	7	6	6	56
	500	6	6	0	6	6	7	0	2	6	6	45
	550	6	6	6	6	6	7	5	7	5	6	60
	600	5	6	4	6	6	7	6	6	6	6	58
C5	450	5	-	6	-	6	-	6	-	6	-	29
	500	6	-	6	-	5	-	6	-	6	-	29
	550	5	-	6	-	6	-	6	-	6	-	29
	600	6	-	6	-	5	-	6	-	6	-	29
C6	400	6	-	7	-	6	-	7	-	7	-	33
	450	6	-	6	-	6	-	7	-	4	-	29
	500	0	-	6	-	6	-	7	-	7	-	26
	550	6	-	5	-	6	-	6	-	6	-	29
O1[ǂ]	400	11	-	11	-	9	-	11	-	9	-	51
	450	10	-	11	-	11	-	11	-	11	-	54
	500	0	-	9	-	10	-	9	-	12	-	40
	550	0	-	10	-	9	-	6	-	10	-	35
O2[ǂ]	350	13	-	12	-	13	-	13	-	13	-	64
	400	12	-	12	-	12	-	12	-	13	-	61
	450	10	-	12	-	10	-	0	-	12	-	44
	500	9	-	11	-	0	-	0	-	7	-	27
O3[ǂ]	350	12	11	8	13	12	12	12	0	12	13	105
	400	11	12	12	13	12	13	12	12	13	13	123
	450	12	12	9	8	10	11	9	6	9	0	86
	500	9	8	8	9	9	9	9	10	0	9	80

MRR design	g_c (nm)	Number of analyzed resonances										
		Chip 1		Chip 2		Chip 3		Chip 4		Chip 5		Total
		Ring 1	Ring 2	Ring 1	Ring 2	Ring 1	Ring 2	Ring 1	Ring 2	Ring 1	Ring 2	
O4	400	9	8	6	7	6	9	6	8	11	7	77
	450	10	5	11	8	9	9	10	11	10	10	93
	500	10	10	10	9	9	10	10	10	10	0	88
	550	10	10	10	10	11	10	11	11	10	0	93

Section S5. Effect of Spatial Location on Group Index

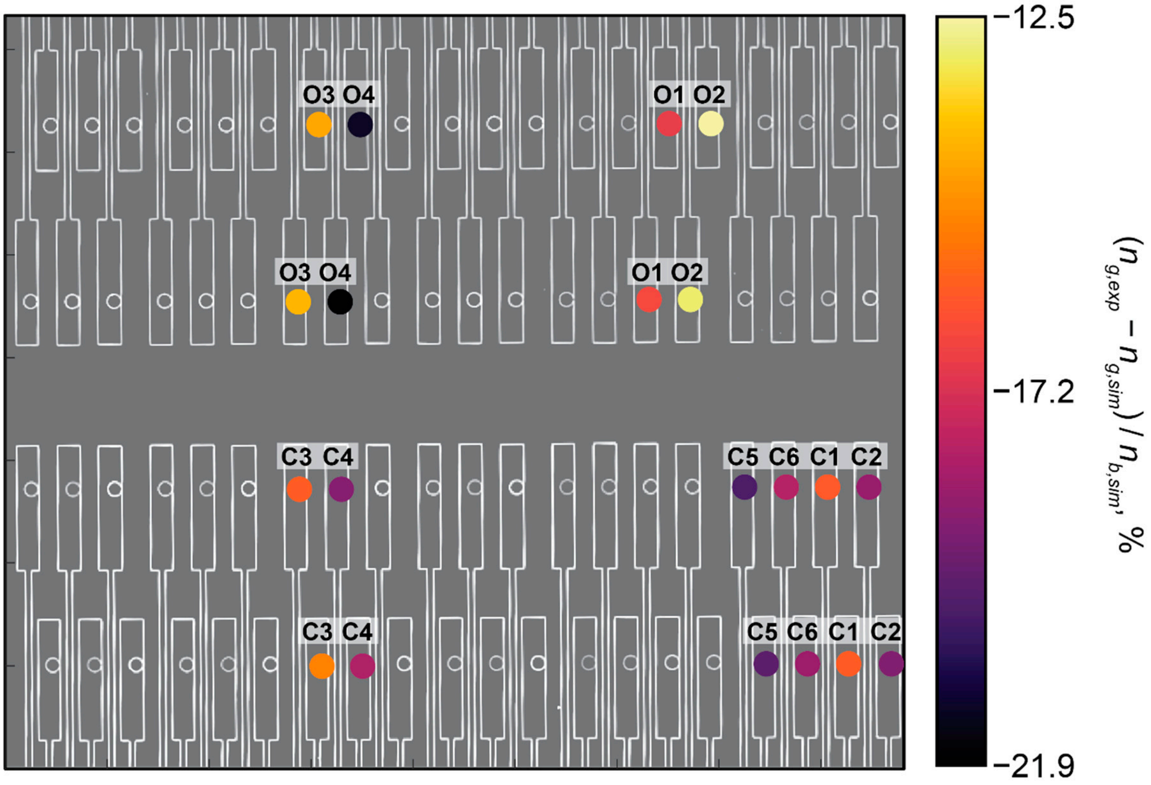

Figure S5. Bright-field micrograph of sensor chip (converted to grayscale) with markers indicating the locations of each ring resonator used for the bulk refractive index sensing measurements. The marker colors are mapped to the percent difference between the measured and simulated n_g for each resonator, based on spectra collected during the bulk refractive index sensing experiments.
